# Adequacy of cool running water first aid by healthcare professionals in the treatment of paediatric burns: A cross‐sectional study of 4537 children

**DOI:** 10.1111/1742-6723.13686

**Published:** 2020-11-15

**Authors:** Cody C Frear, Bronwyn Griffin, Roy Kimble

**Affiliations:** ^1^ Centre for Children's Burns and Trauma Research Centre for Children's Health Research Brisbane Queensland Australia; ^2^ Faculty of Medicine The University of Queensland Brisbane Queensland Australia; ^3^ School of Nursing, Faculty of Health Queensland University of Technology Brisbane Queensland Australia; ^4^ Department of Paediatric Surgery, Urology, Burns and Trauma Queensland Children's Hospital Brisbane Queensland Australia

**Keywords:** burn, emergency, first aid, general practice, paediatric, paramedic

## Abstract

**Objective:**

To determine the adequacy of cool running water first aid provided by healthcare professionals in the early management of children with thermal burn injuries.

**Methods:**

A cross‐sectional study was undertaken using a prospectively collected registry of children who presented with a thermal burn to the only major paediatric burns centre in Queensland, Australia, from January 2013 to December 2018. Main outcome measures included the type and duration of first aid administered by paramedics, general practitioners and emergency providers at local general hospitals and a children's hospital. In accordance with current Australian guidelines, adequate cooling was defined as 20 min of cool running water within 3 h of the injury.

**Results:**

Of the 4537 children who presented to the paediatric burns centre, 3261 (71.9%) received adequate first aid, including 1502 (33.1%) at the scene of injury. Paramedics and general practitioners administered adequate cooling to 184 (25.0%) and 52 (24.2%) of their patients, respectively. ED clinicians adhered to guidelines in the treatment of 1019 (56.3%) children at general hospitals and 411 (76.0%) at the children's hospital. Among ED patients who presented with incomplete prior first aid, the risk of inadequate cooling was significantly greater for those transported via ambulance (*P* < 0.001).

**Conclusion:**

Deficiencies remain in the cooling of paediatric burns patients at all levels of initial management. There is a need in the healthcare community for improved education regarding the parameters and clinical benefits of cool running water first aid.

## Graphical abstract

In the management of burns, first‐aid guidelines recommend the application of 20 min of cool running water up to 3 h following injury. This cross‐sectional study of 4537 paediatric burns patients in Queensland determined that deficiencies remain in the cooling of paediatric burns at all levels of initial care. Adequate cooling was provided to only 25% of children seen by paramedics and general practitioners, 56% of children who presented to a local general hospital and 76% of children treated at the children's hospital.

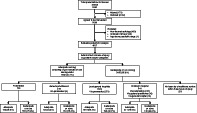

**Key findings**

- In the initial management of paediatric burns, first‐aid guidelines recommend the application of 20 min of cool running water up to 3 h following injury.
- In Queensland, Australia, adequate cooling was provided to only 25% of children seen by paramedics and general practitioners, 56% of children who presented to a local general hospital and 76% of children's hospital patients.
- Among children treated in EDs, the odds of adequate cooling were decreased in those transported via ambulance.

## Introduction

Burns are among the most common form of injury in Australian children, often resulting from domestic accidents involving hot liquids, food and cooking surfaces.^1^, ^2^ Although they account for nearly 4% of all injury‐related hospital admissions,^3^ most paediatric burns are small‐to‐medium‐sized injuries that are managed exclusively on an outpatient basis.^4^ Nonetheless, these wounds carry the potential to cause long‐term sequelae such as scarring,^5^ chronic pain^6^ and sensory disturbances.^7^ Proper initial management of burns, including appropriate first aid, is key to the prevention or minimisation of such complications.^8^

In Australia and New Zealand, first‐aid guidelines recommend the following in the event of a thermal burn injury: (i) extrication from the source of heat; (ii) removal of garments/jewellery from the affected area; (iii) application of cool running water (CRW) for 20 min duration, either consecutively or cumulatively, within 3 h of the injury; and (iv) coverage of the site with a clean cloth or cling wrap.^9^ The administration of CRW not only serves an analgesic function but has been associated with significantly improved patient outcomes, including reduced odds of skin grafting.^10^, ^11^, ^12^ Nevertheless, less than one‐third of Australian children receive adequate CRW from caregivers immediately post‐injury.^13^ The responsibility therefore falls to healthcare professionals to ensure the delivery of appropriate cooling. The purpose of the present study was to evaluate the adequacy of CRW therapy administered by paramedics, general practitioners (GPs) and ED clinicians to paediatric burns patients not given appropriate first aid at the scene of the injury.

## Methods

We undertook a cross‐sectional retrospective study using Queensland Paediatric Burns Registry (QPBR) data for all children under 16 years of age who underwent treatment for a thermal burn from January 2013 to December 2018. The QPBR is a prospectively‐collected database of children presenting to the only major paediatric burns centre in Queensland, Australia. The patients who undergo treatment at the centre are referred from local hospitals and GP clinics throughout Queensland and northern New South Wales.

Data were collected via structured interviews carried out by investigators who approached all families at their initial presentation to the burns service. Families were asked to detail the first aid administered by all parties potentially involved in initial management, including:

1. Caregivers and/or patients at the scene of the injury;
2. Paramedics;
3. GPs;
4. General hospital EDs; and/or
5. The ED of the children's hospital housing the burns centre.

The type of first aid was documented (e.g. CRW, ice, damp cloth), along with the duration of CRW therapy at each level of management, which was divided at the time of collection into five categories:

1. No CRW;
2. Less than 5 min;
3. 5 to 10 min;
4. 11 to 19 min; and
5. Greater than or equal to 20 min.

In addition to documenting the care administered by each individual service or provider, investigators indicated whether patients' overall care satisfied the minimum criteria for adequate cooling.

### Data preparation

Consistent with current guidelines,^9^ cooling was deemed adequate if it involved at least 20 min of CRW within 3 h of the injury. Treatments not adhering to this description, whether consisting of an alternative to or a shorter duration of CRW, were classified as inadequate. We restricted analyses of first aid to care provided in the first 3 h post‐burn. We also excluded non‐thermal injuries such as friction, chemical, electrical and radiation burns, since the first‐aid guidelines are based primarily on studies employing thermal models.

In analyses of individual services, we included all patients with inadequate prior cooling who made contact with a given provider at any point in the 3‐h study window. The length of the cooling administered by a service was added to the cumulative duration of CRW provided up to that point. If a patient received both CRW and an alternative, they were grouped according to the length of their CRW therapy.

In addition to analysing individual services, we classified patients by the full sequence of providers to which they presented (if there was more than one) to compare the adequacy of the care provided by the five most commonly used services or combinations of services:

1. General hospital EDs;
2. Paramedics and general hospital EDs;
3. The children's hospital ED;
4. Paramedics and the children's hospital ED; and
5. GPs.

The focus of this second analysis was the adequacy of the children's cumulative duration of CRW. Patients administered adequate cooling by caregivers and/or the children themselves were excluded.

Each patient was allocated a socio‐economic index for areas (SEIFA)^14^ score, based on their residential postcode. We grouped the SEIFA scores into tertiles (disadvantaged, advantaged, and highly advantaged). Accessibility/Remoteness Index of Australia (ARIA+)^15^ scores were assigned to injury locations. These scores were collapsed into two levels – metropolitan (major cities) and non‐metropolitan (inner regional, outer regional, remote and very remote) – because of the small sample sizes of the outer regional, remote and very remote categories.

### Post hoc *review*

As the QPBR did not capture the presence of any other injuries sustained by patients in addition to their burns, a *post hoc* chart review of 100 randomly selected cases was conducted to estimate the proportion of children affected by multiple trauma.

### Statistical methods

Descriptive statistics were calculated for all key variables. Median and interquartile range were reported for age and total body surface area percentage affected, which both conformed to a non‐normal distribution. Differences in these data were evaluated in a univariable fashion using Kruskal‐Wallis and Mann–Whitney tests. Where variables were purely categorical, χ^2^‐tests were employed.

Logistic regression models were fitted to assess the relationship between several patient‐ and injury‐level characteristics and the delivery of adequate first aid by individual providers. They were also employed to compare CRW adequacy between the five most commonly used services or combinations of services. Variables were included in the final multivariable models if they produced a *P*‐value of <0.05 in the univariable analyses. Statistical analyses were performed using SPSS v. 25 (SPSS Inc., Chicago, IL, USA). A *P*‐value of <0.05 was considered statistically significant.

### Ethics approval

Ethics approval for the QPBR was granted by the Children's Health Service District – Human Research Ethics Committee (approval no: HREC/16/QRCH/61).

## Results

During the study period, 5293 children presented to the paediatric burns centre. Of these, 173 were missed and 32 declined to be interviewed. A further 120 were excluded because of unknown or unclear first aid, 430 for non‐thermal aetiology and one for falling outside the paediatric age range.

Demographic details for the remaining 4537 are provided in Table 1. Overall, 3261 (71.9%) received adequate cooling. At the scene of the incident, caregivers and/or the patients themselves provided adequate CRW in 1502 (33.1%) cases. For 2223 (49.0%) children, cooling with running water was initiated but not completed. The remainder of the cohort was administered either no first aid (311; 6.9%) or an alternative to CRW (500; 11.0%). Ice was employed by 593 (13.1%) caregivers.

**TABLE 1:** Patient and injury demographics by healthcare provider

Variable	All patients (*n* = 4537)	Paramedics (*n* = 735)	General practitioners (*n* = 215)	General hospitals (*n* = 1809)	Children's hospital (*n* = 541)
Patient age (years), median (IQR)	2 (1–6)	2 (1–6)	1 (1–5)	2 (1–6)	2 (1–5)
Total body surface area percentage affected, median (IQR)	1 (1–2)	1.5 (1–3)	1 (1–1.5)	1 (1–2)	1 (1–2)
Patient sex, *n* (%)					
Male	2662 (58.9)	423 (58.1)	133 (62.1)	1078 (59.8)	295 (55.0)
Indigenous status, *n* (%)					
Aboriginal or Torres Strait Islander	388 (9.4)	77 (11.2)	10 (5.0)	186 (11.1)	32 (6.3)
Socio‐economic status, *n* (%)					
Disadvantaged	1360 (30.7)	232 (32.4)	60 (28.4)	671 (37.8)	108 (20.3)
Advantaged	1802 (40.6)	295 (41.3)	82 (38.9)	754 (42.5)	171 (32.2)
Highly advantaged	1271 (28.7)	188 (26.3)	69 (32.7)	348 (19.6)	252 (47.5)
Region of injury, *n* (%)					
Metropolitan	3766 (86.8)	619 (88.1)	189 (91.7)	1379 (80.5)	510 (96.6)
Non‐metropolitan	571 (12.6)	84 (11.9)	17 (8.3)	333 (19.5)	18 (3.4)
Mechanism of injury, *n* (%)					
Scald	2266 (49.9)	515 (70.1)	93 (43.3)	825 (45.6)	349 (64.5)
Contact	2049 (45.2)	168 (22.9)	110 (51.2)	889 (49.1)	173 (32.0)
Flame	220 (4.8)	51 (6.9)	12 (5.6)	94 (5.2)	19 (3.5)
Radiant heat	2 (<0.1)	1 (0.1)	0 (0.0)	1 (<0.1)	0 (0.0)
Place of injury, *n* (%)					
Home	3844 (84.7)	619 (85.3)	181 (85.4)	1506 (84.5)	471 (88.4)
Holiday	277 (6.2)	43 (5.9)	12 (5.7)	129 (7.2)	12 (2.3)
Industrial/trade/farm	78 (1.7)	15 (2.1)	3 (1.4)	35 (2.0)	12 (2.3)
Recreation/sports	147 (3.2)	26 (3.6)	6 (2.8)	73 (4.1)	18 (3.4)
School or residential	75 (1.7)	12 (1.7)	7 (3.3)	18 (1.0)	17 (3.2)
Street	21 (0.5)	7 (1.0)	2 (0.9)	11 (0.6)	1 (0.2)
Other	21 (0.5)	4 (0.6)	1 (0.5)	10 (0.6)	2 (0.4)
Body part injured, *n* (%)					
Upper limb	1802 (40.3)	157 (21.8)	109 (51.4)	696 (39.1)	179 (33.6)
Multiple	1062 (23.7)	315 (43.7)	24 (11.3)	423 (23.8)	171 (32.1)
Lower limb	1048 (23.4)	127 (17.6)	54 (25.5)	468 (26.3)	84 (15.8)
Torso	396 (8.9)	94 (13.0)	13 (6.1)	127 (7.1)	70 (13.2)
Head	164 (3.7)	28 (3.9)	12 (5.7)	67 (3.8)	28 (5.3)
Total body surface area percentage affected, *n* (%)					
<5	4112 (91.0)	583 (79.4)	204 (94.9)	1661 (92.1)	476 (88.5)
5–10	281 (6.3)	92 (12.6)	10 (4.6)	96 (5.3)	44 (8.2)
>10	124 (2.7)	59 (8.0)	1 (0.5)	46 (2.6)	18 (3.3)
Wound depth, *n* (%)					
Superficial	172 (3.9)	17 (2.3)	6 (2.8)	66 (3.7)	23 (4.3)
Superficial partial‐thickness	3005 (67.3)	458 (62.9)	140 (66.0)	1116 (62.7)	390 (73.3)
Deep dermal partial‐thickness	1117 (25.0)	210 (28.8)	56 (26.4)	505 (28.4)	111 (20.9)
Full thickness	168 (3.8)	43 (5.9)	10 (4.7)	93 (5.2)	23 (4.3)

Full cohort data were complete for age and mechanism. Categories in which data were missing included: total body surface area percentage affected (*n* = 20, 0.44%); sex (*n* = 21, 0.46%); indigenous status (*n* = 395, 8.71%); socio‐economic status (*n* = 104, 2.29%); region of injury (*n* = 200, 4.41%); place of injury (*n* = 74, 1.63%); body part injured (*n* = 65, 1.12%) and wound depth (*n* = 75, 1.65%).

IQR, interquartile range.

**Figure 1:**
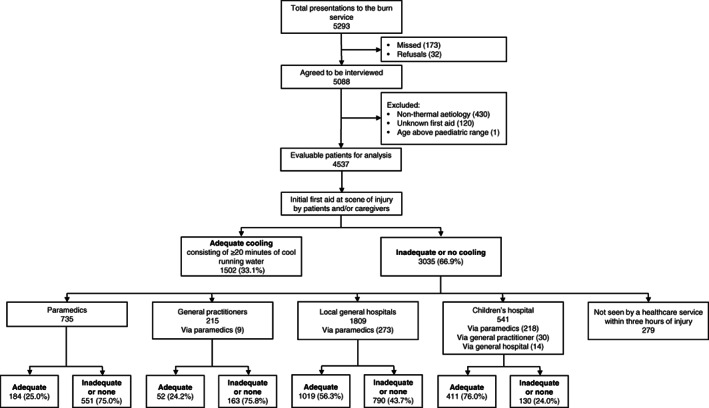
Flow of participants and first aid outcomes. The statistics for the individual providers encompass all patients who presented to a given service in the first 3 h of their injury following previously inadequate first aid, including those who presented to multiple services.

**Figure 2:**
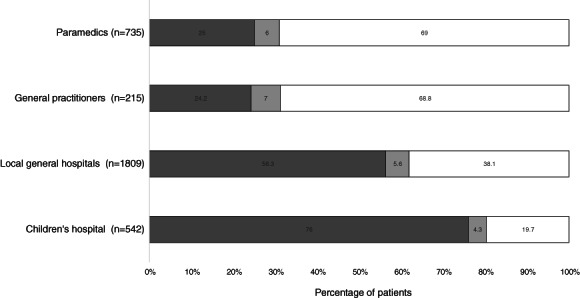
Adequacy of cool running water first aid by healthcare professionals. (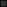), Adequate cooling; (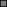), inadequate cooling; (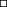), no cooling with running water.

Among the 3035 children not given adequate initial CRW first aid, 2756 (90.8%) were subsequently treated by paramedics, a GP, a general hospital ED, the children's hospital ED or some combination thereof up to 3 h after the burn. As depicted in Figure 1, inadequate cooling was rectified in 184 (25.0%, 95% confidence interval [CI] 22–28%) of the 735 patients with deficient prior first aid seen by paramedics, 52 (24.2%, 95% CI 18–30%) of the 215 who presented to a GP, 1019 (56.3%, 95% CI 54–59%) of the 1809 treated by a general hospital and 411 (76.0%, 95% CI 72–80%) of the 541 managed in the children's hospital. A total of 383 children (12.6%) were not administered water cooling by any healthcare professionals involved in their care (Fig. 2). Other forms of first aid employed by paramedics included hydrogels (e.g. BurnAid®), which were provided to 50 (6.8%) patients in addition to water and to 201 (27.3%) as a substitute for it.

Several patient‐ and injury‐level factors were analysed to assess their potential relationship with the provision of adequate cooling (Table S1). There were no associations in the multivariable models with sex, ethnicity, anatomical location or total body surface area. Paramedics and general hospitals showed reduced odds of administering adequate CRW to contact burns and injuries occurring in non‐metropolitan locations, though their overall odds of adhering to guidelines appeared to improve over time. Among children who presented to the children's hospital with incomplete or no prior first aid, adequate cooling was provided to 148 (67.9%) of the 218 brought in by ambulance and 263 (81.4%) of the 323 transported via other means (odds ratio [OR] 0.51, 95% CI 0.34–0.78, *P* < 0.001). The discrepancy was even greater in general hospitals: 106/273 (38.8%) *versus* 913/1536 (59.4%, OR 0.41, 95% CI 0.31–0.54, *P* < 0.001).

**Figure 3:**
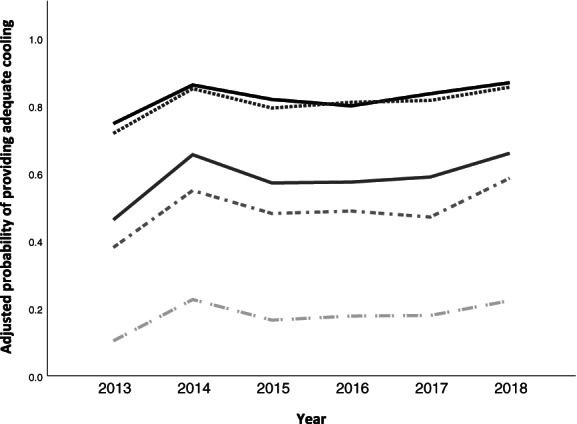
Probability of adequate first aid delivery by the five most commonly used services or combinations of services over the study period. Values were obtained using logistic regression models adjusting for mechanism of injury, socio‐economic status and location. Healthcare service: (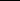), children's hospital (*n* = 279); (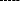), paramedics and children's hospital (*n* = 218); (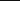), general hospitals (*n* = 1536); (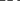), paramedics and general hospitals (*n* = 273); (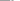), general practitioners (*n* = 206).

As these results did not take into account the provision of adequate cooling by paramedics, we also compared overall CRW adequacy across different combinations of services (Fig. 3). Adjusting for mechanism of injury, socioeconomic advantage, and accessibility/remoteness, direct presentation to hospital was associated with significantly greater odds of adequate CRW than combined paramedic and ED services for general hospitals (828/1411 [58.7%] *vs* 153/310 [49.4%], OR 1.57, 95% CI 1.20–2.04, *P* = 0.001), but not for the children's hospital (257/310 [82.9%] *vs* 293/364 [80.5%], OR 1.19, 95% CI 0.79–1.80, *P* = 0.404).

A *post hoc* chart review revealed that, in a random sample of 100 patients, only one case of multiple trauma was documented.

## Discussion

The present study demonstrates deficiencies in the cooling provided by all healthcare services involved in the initial management of paediatric burns patients. Approximately 75% of the children seen by paramedics and GPs failed to receive 20 min of CRW as recommended by the Australian and New Zealand Burn Association.^9^ EDs were generally more likely to follow current guidelines, although first aid was still lacking for 24% and 44% of patients treated at the children's hospital and general hospitals, respectively.

Despite signalling a clear need for further education in the healthcare community regarding the importance of CRW first aid, these findings represent a substantial improvement from the practices documented in previous research. In a 2001 study^16^ involving 109 children, a children's hospital in Sydney adhered to first‐aid guidelines in the treatment of under 36% of its burns patients, with even lower levels of adherence among general hospitals (29%) and GP clinics (0%). In a 2014 analysis of 117 paediatric patients in Queensland,^17^ cooling with running water was provided by paramedics in 11% of cases. Even in surveys, clinicians exhibit only moderate to fair knowledge of optimal first aid, with 30–40% failing to correctly identify the appropriate initial management for a paediatric scald on a multiple‐choice questionnaire.^18^, ^19^

Much of the progress in first‐aid awareness and practice is likely related to research that better defined the parameters and benefits of optimal first aid,^20^, ^21^, ^22^ which in turn prompted increased efforts by Queensland's paediatric burns team to promote CRW first aid when contacted by referral centres. Recent updates to Queensland Health's online resources^23^ and the Queensland Ambulance Service's guidelines^24^ reflect a greater emphasis on cooling for the recommended duration. Nevertheless, in a high‐income country such as Australia, where clean running water is widely available, it is remarkable that appropriate first aid remains so elusive for large numbers of children. Undoubtedly, burns can and do occur in areas without immediate access to running water, presenting obvious challenges to any ambulance services attending the scene. It may be for this reason that paramedics were less likely to administer adequate CRW to contact burns than scalds; a higher proportion of contact burns occurred in outdoor settings (e.g. camp sites). Such cases, however, comprised a small minority of children seen by paramedics, more than 85% of whom sustained their burns in the home.

The finding that children brought to hospital by ambulance were less likely to receive first aid than patients who presented directly to an ED merits further investigation. One possible explanation may be a lack of clear communication between providers. Ensuring that first aid is prioritised as a critical element of handover may help address this issue. Alternatively, the pharmacological analgesia administered by ambulance services might produce differences in pain between the two groups. In patients with better‐controlled pain, there might be less of an impetus for clinicians to provide CRW if they believe its role is analgesic.

The wider clinical benefits associated with adequate cooling beyond pain management have been well demonstrated in multiple human studies. We previously found that children given adequate CRW exhibited reduced odds of skin grafting, full‐thickness depth, hospital admission and theatre operations.^12^ Similar research in adults suggested first aid may lead to faster re‐epithelialisation,^11^ reduced hospital length of stay and decreased intensive care admission requirements.^10^ By comparison, the use of hydrogels, which ambulance services administered to one‐quarter of their patients in lieu of CRW, is not supported by existing evidence that shows they have no significant effect on wound healing.^25^

Some healthcare professionals may be concerned about the possibility of hypothermia, which, among children especially, is a legitimate risk that can adversely affect patient outcomes.^26^ It is partly for this reason that ice, which was used by 13% of caregivers, is heavily discouraged as a form of first aid. Even when hypothermia is not a risk, ice provides no observable benefit to burn healing and may in fact exacerbate tissue damage.^21^ If appropriate steps are taken, however, fear of hypothermia should not deter healthcare professionals from providing adequate first aid to paediatric burns, the majority of which can be targeted with isolated cooling while the rest of the child is warmed. In larger injuries, for which there is evidence to suggest cooling provides significant clinical benefits,^27^ it is still possible to protect against hypothermia by delivering cool (not cold) water and allowing patients to take breaks if their temperature begins to drop.

### Limitations

The study relied on data obtained via structured interviews conducted with families at their first presentation to the burns clinic, which inherently posed a risk of recall bias. In retrospective reporting, the duration of stressful events tends to be inflated,^28^ potentially overestimating the proportion of children administered adequate first aid. However, a previous study using the same data registry noted substantial concordance between interviews and contemporaneous clinical records.^12^

In scenarios where severe multiple trauma is common (e.g. house fires), other issues could demand more urgent attention than a child's burns. The interviews did not include questions explicitly pertaining to this possibility. Nevertheless, a *post hoc* chart review suggested the vast majority of cases involved isolated burns without any concomitant injuries that would render cooling a secondary consideration.

## Conclusion

The initial care provided by most GPs and paramedics, as well as many ED clinicians, fell short of current guidelines calling for 20 min of cooling with running water. Among children treated at hospitals, the odds of adequate cooling were decreased in those transported via ambulance. These findings highlight the need for improved communication of cooling during handover, campaigns to increase the public's awareness of appropriate first aid, and greater education of nursing, paramedic, and medical students regarding the therapy's parameters and associations with improved outcomes. In recent years, haphazard approaches have yielded small gains, but the cultivation of widespread adherence to guidelines will require a planned implementation process.

### Author contributions

CCF, BG and RK conceived and designed the study. BG and RK supervised data collection. CCF performed the analysis. BG and RK contributed to interpretation of the results. CCF wrote the manuscript with input from BG and RK.

### Competing interests

None declared.

## Supporting information


**Table S1.** Factors associated with the provision of adequate first aid to children with no or inadequate prior cooling: logistic regression analyses.

## Data Availability

The data that support the findings of this study are available from the corresponding author upon reasonable request.
