# Supplementary material for: Adequacy of cool running water first aid by healthcare professionals in the treatment of paediatric burns: A cross‐sectional study of 4537 children
Source: Emerg Med Australas. 2020 Nov 15;33(4):615–22. doi: 10.1111/1742-6723.13686 (PMC9292905; doi:10.1111/1742-6723.13686)
Supplement: Supplementary file 1 — Table S1. Factors associated with the provision of adequate first aid to children with no or inadequate prior cooling: logistic regression analyses. [file EMM-33-615-s001.docx]

***Supplementary Table 1. Factors associated with the provision of adequate first aid to children with no or inadequate prior cooling*: *logistic regression analyses***

|  |  | Paramedics | | General practitioners | | General hospitals | | Children’s hospital | |
| --- | --- | --- | --- | --- | --- | --- | --- | --- | --- |
|  | | OR (95% CI) | Adjusted OR (95% CI) | OR (95% CI) | Adjusted OR (95% CI) | OR (95% CI) | Adjusted OR (95% CI) | OR (95% CI) | Adjusted OR (95% CI) |
| Age |  | 0.96 (0.92-1.00) | - | 0.97 (0.89-1.05) | - | 0.97 (0.95-0.99) | - | 0.99 (0.95-1.04) | - |
| Sex | Female | 1.01 (0.72-1.42) | - | 0.74 (0.39-1.44) | - | 1.13 (0.93-1.36) | - | 1.16 (0.78-1.73) | - |
| TBSA |  | 1.01 (0.98-1.03) | - | 1.14 (0.93-1.39) | - | 0.99 (0.97-1.02) | - | 0.95 (0.90-1.00) | - |
| Indigenous status | Indigenous | 0.97 (0.56-1.68) | - | 0.73 (0.15-3.58) | - | 0.76 (0.56-1.04) | - | 0.78 (0.35-1.73) | - |
| Mechanism^†^ | Scald | 1 (Ref.) | 1 (Ref.) | 1 (Ref.) | - | 1 (Ref.) | 1 (Ref.) | 1 (Ref.) | - |
|  | Contact | 0.31 (0.18-0.51) | 0.28 (0.16-0.48) | 0.89 (0.47-1.66) | - | 0.67 (0.55-0.81) | 0.58 (0.47-0.71) | 0.91 (0.60-1.39) | - |
|  | Flame | 0.83 (0.43-1.59) | - | ^‡^ | ^‡^ | 0.71 (0.46-1.08) | - | 0.65 (0.24-1.77) | - |
| Body site | Upper limb | 1 (Ref.) | - | 1 (Ref.) | - | 1 (Ref.) | - | 1 (Ref.) | - |
|  | Multiple sites | 1.23 (0.78-1.93) | - | 1.60 (0.61-4.15) | - | 0.97 (0.76-1.23) | - | 0.69 (0.42-1.12) | - |
|  | Lower limb | 0.82 (0.46-1.47) | - | 0.91 (0.42-1.99) | - | 0.76 (0.60-0.97) | - | 0.80 (0.44-1.48) | - |
|  | Torso | 1.72 (0.97-3.04) | - | 1.42 (0.40-4.99) | - | 1.11 (0.75-1.63) | - | 0.98 (0.50-1.93) | - |
|  | Head | 1.65 (0.69-3.97) | - | 0.29 (0.04-2.36) | - | 0.83 (0.50-1.38) | - | 0.98 (0.37-2.59) | - |
| Year | 2013 | 1 (Ref.) | 1 (Ref.) | 1 (Ref.) | - | 1 (Ref.) | 1 (Ref.) | 1 (Ref.) | - |
|  | 2014 | 1.23 (0.62-2.42) | - | 2.09 (0.62-7.10) | - | 1.61 (1.15-2.26) | 1.69 (1.17-2.44) | 1.71 (0.74-3.92) | - |
|  | 2015 | 1.12 (0.56-2.24) | - | 1.58 (0.49-5.07) | - | 1.19 (0.85-1.68) | - | 0.89 (0.44-1.83) | - |
|  | 2016 | 2.46 (1.30-4.66) | 2.42 (1.25-4.67) | 1.84 (0.55-6.19) | - | 1.30 (0.93-1.82) | - | 0.84 (0.41-1.72) | - |
|  | 2017 | 1.86 (0.95-3.65) | 2.57 (1.27-5.20) | 1.29 (0.36-4.63) | - | 1.51 (1.09-2.11) | 1.59 (1.11-2.26) | 0.90 (0.43-1.89) | - |
|  | 2018 | 3.30 (1.67-6.50) | 3.84 (1.89-7.81) | 1.12 (0.32-3.85) | - | 2.14 (1.52-3.01) | 2.12 (1.48-3.05) | 1.34 (0.61-2.95) | - |
| Socio-economic advantage | Highly advantaged | 1 (Ref.) | - | 1 (Ref.) | - | 1 (Ref.) | 1 (Ref.) | 1 (Ref.) | 1 (Ref.) |
|  | Advantaged | 0.78 (0.52-1.18) | - | 0.93 (0.43-2.00) | - | 0.67 (0.52-0.87) | 0.74 (0.57-0.98) | 0.91 (0.57-1.46) | - |
|  | Disadvantaged | 0.73 (0.47-1.13) | - | 1.31 (0.59-2.89) | - | 0.71 (0.55-0.93) | - | 0.50 (0.30-0.82) | 0.59 (0.35-0.99) |
| Presentation | Via ambulance | - | - | ^‡^ | ^‡^ | 0.43 (0.33-0.56) | 0.41 (0.31-0.54) | 0.48 (0.32-0.72) | 0.53 (0.35-0.80) |
| Region of injury | Non-metropolitan | 0.29 (0.14-0.61) | 0.24 (0.11-0.53) | 0.65 (0.18-2.35) | - | 0.73 (0.57-0.93) | 0.73 (0.56-0.95) | ^‡^ | ^‡^ |

Adjusted OR only provided for variables yielding a *P*-value of <0.05 in the univariable analyses. ^†^Radiant heat burns (n=3) were excluded. ^‡^Insufficient in number to be included in the model.
